# Multidrug‐Resistant Anal and Perianal Tuberculosis: A Case Report From the Pneumo‐Phthisiology Department of Conakry University Hospital, Guinea

**DOI:** 10.1155/crpu/3497569

**Published:** 2026-02-24

**Authors:** Oumou Hawa Diallo, Mamadou Hawa Camara, Boubacar Djelo Diallo, Thierno Mouctar Bah, Ousmane N′Namarie Camara, Aboubacar Camara, Lansana Mady Camara

**Affiliations:** ^1^ Faculty of Health Sciences and Techniques, Gamal Abdel Nasser University of Conakry, Conakry, Guinea, uganc.org; ^2^ Department of Pneumo-Phthisiology, Ignace Deen University Hospital Center, Conakry, Guinea

**Keywords:** anal tuberculosis, Conakry, extrapulmonary tuberculosis, Guinea, Ignace Deen Hospital, multidrug-resistant tuberculosis

## Abstract

**Introduction:**

Multidrug‐resistant (MDR) anal and perianal tuberculosis constitutes an exceptionally rare form of extrapulmonary tuberculosis. We report a case of MDR anal and perianal tuberculosis diagnosed and managed in the Pneumo‐Phthisiology Department of Ignace Deen University Hospital in Conakry, Guinea.

**Case Presentation:**

Mrs. M.B., a 33‐year‐old housewife residing in Tombolia (Conakry), with no notable medical history, presented to the General Surgery Department of Ignace Deen University Hospital on 24 February 2023 with fever, abdominal pain, constipation, and painful swelling of the anal and perianal region. Following a hemorrhoidectomy, histopathological examination of the surgical specimen initially suggested a diagnosis of diffuse large B‐cell lymphoma of the anal region. Consequently, CHOP chemotherapy (Adriamycin, cyclophosphamide, vincristine, and prednisolone) was initiated on 10 March 2023 in the Hematology Department. After three cycles of chemotherapy, the patient showed no clinical improvement, with persistent anal lesions and recurrent fever. A strongly positive tuberculin skin test (15 mm) prompted referral to the Pneumo‐Phthisiology Department for suspected anal tuberculosis. GeneXpert MTB/RIF testing performed on stool samples confirmed the presence of MDR *Mycobacterium tuberculosis*. A 9‐month short‐course second‐line antituberculosis regimen was initiated. After 1 month of treatment, the patient developed abdominal pain, semiliquid diarrhea, anorexia, and abdominal distension with a positive fluid‐thrill sign. The anal and perianal lesions, however, showed significant improvement.

**Conclusion:**

MDR anal and perianal tuberculosis is an uncommon manifestation of extrapulmonary tuberculosis. In regions with high tuberculosis endemicity, it should be considered in the differential diagnosis of chronic ulcerative cutaneous or mucosal lesions. Management relies primarily on second‐line antituberculosis therapy to prevent complications and ensure complete recovery.

## 1. Introduction

Significant progress has been made in recent years in the diagnosis of tuberculosis (TB) in low‐ and middle‐income countries. This shift followed the recognition that reliance on acid‐fast bacilli smear microscopy alone was insufficient to address the dual challenges posed by human immunodeficiency virus (HIV)–associated TB and drug‐resistant TB—two threats capable of reversing global TB‐control achievements [1].

TB remains one of the leading causes of morbidity and mortality worldwide. According to the World Health Organization (WHO), 10.8 million new TB cases were reported in 2023, with 1.25 million associated deaths [2]. Although TB predominantly affects the lungs, extrapulmonary involvement can occur at virtually any anatomical site [3, 4]. Extrapulmonary drug‐resistant tuberculosis (DR‐EPTB) presents substantial diagnostic and therapeutic difficulties [5]. Extrapulmonary TB represents less than 15% of all TB cases, and intestinal TB accounts for fewer than 1% of extrapulmonary cases [6, 7].

Early diagnosis of intestinal TB—particularly involving the anal and perianal regions—remains challenging due to its nonspecific presentation, which frequently mimics other gastrointestinal disorders. Anal and perianal TB are extremely rare, with only a limited number of tuberculous anal fistulas documented in the literature [8, 9].

Malignant lymphomas, characterized by clonal proliferation of mature B or T lymphocytes, may initially mimic chronic infectious diseases, including lymph node TB. However, anal and perianal presentations of lymphoma are rare.

Here, we report a case of multidrug‐resistant anal and perianal TB in a young, HIV‐negative woman hospitalized in the Pneumo‐Phthisiology Department of Ignace Deen University Hospital Center.

## 2. Clinical Case

Mrs. M.B., a 33‐year‐old housewife residing in Tombolia (Conakry) with no significant past medical history, presented to the General Surgery Department of Ignace Deen University Hospital on 24 February 2023 with a 2‐month history of fever, abdominal pain, constipation, and chronic anal and perianal swelling. The initial differential diagnoses included external hemorrhoids and a rectal tumor. A hemorrhoidectomy was performed, and the specimen was submitted for histopathological analysis, which reported diffuse large‐cell lymphoma of the anal and perianal region.

She was referred to the Hematology Department for further evaluation. Laboratory investigations revealed the following: hemoglobin 10.2 g/dL, leukocytes 6.2 × 10^9^/*L*, platelets 498 × 10^3^/*m* *m* ^3^, mean corpuscular volume 84.5 fL, mean corpuscular hemoglobin 24.4 pg, mean corpuscular hemoglobin concentration 23.9 pg, hematocrit 35.3%, and lymphocytes 19.5%; HIV serology negative and hepatitis B surface antigen negative; blood group A Rh positive; serum creatinine 50.33 *μ*mol/L (creatinine clearance 157.76 mL/min); lactate dehydrogenase (LDH) 330 *μ*mol/L; and blood glucose 4.07 *μ*mol/L.

CHOP chemotherapy (Adriblastin 50 mg, cyclophosphamide 750 mg, vincristine 1.4 mg, and prednisolone 40 mg) was initiated. The clinical course was unfavorable, with chemotherapy‐induced vomiting, scalp alopecia, persistent anal and perianal lesions, fever, and progressive weight loss after three cycles. Given the lack of improvement, an intradermal tuberculin skin test (TST) was performed and was strongly positive (15 mm), prompting referral to the Pneumo‐Phthisiology Department for suspected TB.

**Figure 1:**
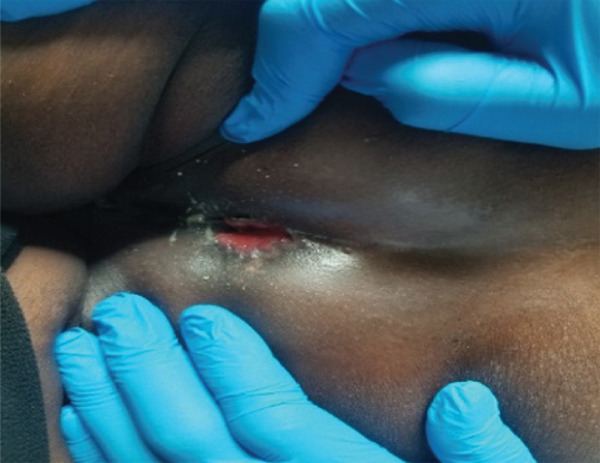
Perianal area at 2 months 14 days postoperatively (M0) at the time of diagnosis of multidrug‐resistant anal and perianal tuberculosis.

Upon admission, the patient weighed 60 kg, measured 170 cm (BMI 20.7 kg/m^2^), and had a temperature of 37.8°C, pulse 104 beats/min, respiratory rate 24 breaths/min, and blood pressure 98/65 mmHg. She had a family history of TB and no toxic habits. Proctologic examination revealed a superficial anal wound with yellowish serous discharge (Figure 1). Respiratory examination was unremarkable. Microbiological testing confirmed TB: Stool Xpert MTB/RIF detected *Mycobacterium tuberculosis* resistant to rifampicin, and a line probe assay (LPA) identified resistance to both rifampicin and isoniazid. Sputum Xpert MTB/RIF was negative for acid‐fast bacilli, and chest radiography was normal. Abdominal and pelvic ultrasound showed multiple intra‐abdominal lymphadenopathies of variable size in the coeliomesenteric and para‐aortic regions without ascites. A second‐line short‐course anti‐TB regimen for multidrug‐resistant anal and perianal TB was initiated according to national guidelines: bedaquiline, high‐dose isoniazid, moxifloxacin, prothionamide, clofazimine, ethambutol, and pyrazinamide (Bdq, Hhn, Mfx, Pto, Cfz, E, and Z) for 9 months, structured as 4 months of Bdq–Pto–Mfx–Cfz–Hhn–E–Z followed by 5 months of Mfx–Cfz–E–Z.

After 1 month of treatment, the patient developed abdominal pain, semiliquid diarrhea (five episodes/day), anorexia, and abdominal distension with a positive fluid‐thrill sign. The anal and perianal lesions, however, showed significant improvement. Stool culture isolated *Escherichia coli* sensitive to ciprofloxacin. Abdominopelvic ultrasound revealed multiple intra‐abdominal lymphadenopathies and moderate ascites. LDH had increased to 484 IU/L (normal 190–445). Xpert MTB/RIF on ascitic fluid was negative, and cytological examination was noncontributory. Management included rehydration (Ringer′s lactate 2 bags/day and 0.9% saline 2 bags/day for 6 days), Nutrisan infusion, oral ciprofloxacin 500 mg twice daily, and folic acid–iron supplementation (one tablet twice daily). Nutritional rehabilitation with wheat‐based WFP flour (three meals/day) and repeated therapeutic paracenteses were also provided.

**Figure 2:**
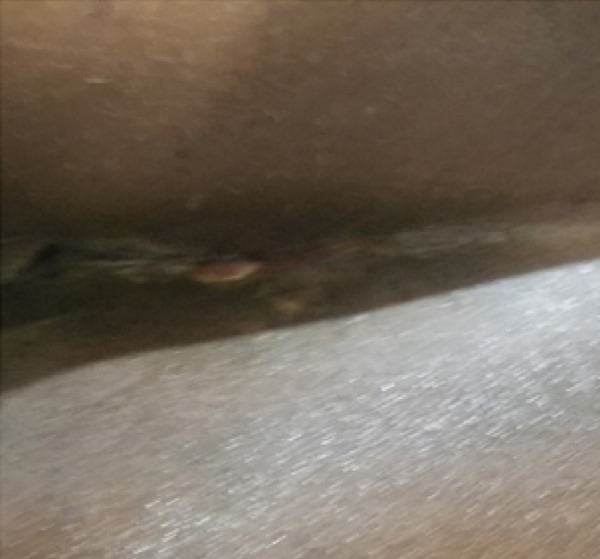
Perianal region at 4 months of second‐line tuberculosis treatment.

At Month 3 of treatment, she developed intercostal herpes zoster. Dermatology consultation led to initiation of oral acyclovir 200 mg (two tablets twice daily) and topical acyclovir ointment twice daily. By Month 4 (15 September 2023), the cutaneous lesions had healed, and her appetite had improved. Clinical evolution was favorable, with progressive weight gain: 65 kg at Month 5, 67 kg at Month 6, 68 kg at Month 7, 69 kg at Month 8, and 70 kg at the end of Month 9. The patient was declared cured after completing treatment, with complete healing of the anal and perianal lesions (Figures 1 and 2).

## 3. Discussion

TB remains a major public health concern in developing countries, including Guinea. In 2023, the country reported 20,011 cases of all forms of TB, corresponding to an incidence of 175 per 100,000 population [10]. Anal and perianal TB are exceptionally rare manifestations of extrapulmonary TB. Most reported cases occur in the context of active pulmonary disease; however, our patient presented with an isolated anal and perianal localization, with no clinical, radiological, or bacteriological evidence of pulmonary involvement. The true prevalence of anal and perianal TB is unknown but is estimated to represent 0.3%–16% of anal fistulas depending on the series [9]. This condition predominantly affects young adult males aged 20–40 years, particularly those who are immunocompromised, most commonly due to HIV infection [8, 9, 11]. In contrast, our patient was a young HIV‐negative woman. Diagnostic confirmation of anal TB is often challenging because the disease is rarely considered initially. Extrapulmonary TB frequently mimics common infections or inflammatory conditions of the anorectal region, resulting in significant diagnostic delays [12]. The clinical manifestations overlap with those of other anal disorders, including Crohn′s disease, chronic suppurative infections, and anal carcinoma [13]. Pathophysiologically, gastrointestinal involvement by *Mycobacterium tuberculosis* may result from ingestion of infected sputum, hematogenous dissemination, or reactivation of latent infection within regional lymph nodes [7]. Although several diagnostic tools are available, early diagnosis remains difficult because there is no pathognomonic clinical sign, and the presentation may be ulcerative, fistulous, or nodular [8]. In our case, the initial histopathological analysis suggested diffuse large‐cell lymphoma, and bacteriological examination of the surgical specimen was not performed. The gold standard for diagnosis remains the identification of epithelioid granulomas with multinucleated giant cells and caseous necrosis on histological examination of fistula edges or excised tissue [14]. However, molecular tests such as Xpert MTB/RIF have revolutionized diagnostic capacity, enabling rapid detection of *Mycobacterium tuberculosis* and rifampicin resistance [12, 15–17]. In our patient, confirmation of MDR TB was obtained through stool PCR testing, which may reflect ingestion of infected secretions even in the absence of radiographic pulmonary involvement [18]. The development of herpes zoster lesions and ascites during treatment may be attributed to immunosuppression secondary to prior chemotherapy, which is known to increase susceptibility to opportunistic infections and viral reactivation.

Overall, this case illustrates the diagnostic difficulty of anal and perianal TB, particularly in atypical presentations and in the absence of pulmonary disease. The delay in diagnosis resulted in unnecessary chemotherapy and contributed to clinical deterioration.

## 4. Conclusion

Multidrug‐resistant anal and perianal TB is a rare form of extrapulmonary TB. In regions where TB is highly endemic, clinicians should consider this diagnosis in any chronic ulcerative or fistulous lesion of the anal or perianal region. Early recognition is essential to prevent misdiagnosis and inappropriate treatment. Management is primarily medical and relies on second‐line anti‐TB therapy according to national and WHO recommendations to avoid complications and ensure complete recovery.

## Funding

No funding was received for this manuscript.

## Consent

Informed oral consent was obtained from the patient for publication of this case study and any accompanying images or data.

## Conflicts of Interest

The authors declare no conflicts of interest.

## Data Availability

The data that support the findings of this study are available from the corresponding author upon reasonable request.
